# Not sold here: limited access to legally available syringes at pharmacies in Tijuana, Mexico

**DOI:** 10.1186/1477-7517-8-13

**Published:** 2011-05-24

**Authors:** Robin A Pollini, Perth C Rosen, Manuel Gallardo, Brenda Robles, Kimberly C Brouwer, Grace E Macalino, Remedios Lozada

**Affiliations:** 1 Division of Global Public Health, Department of Medicine, University of California San Diego, La Jolla, CA, USA; 2 Patronato Pro-COMUSIDA, Tijuana, Mexico; 3 Infectious Disease Clinical Research Program, Department of Preventive Medicine and Biometrics, Uniformed Services University of the Health Sciences, Bethesda, MD, USA

## Abstract

**Background:**

Sterile syringe access is a critical component of HIV prevention programs. Although retail pharmacies provide convenient outlets for syringe access, injection drug users (IDUs) may encounter barriers to syringe purchase even where purchase without a prescription is legal. We sought to obtain an objective measure of syringe access in Tijuana, Mexico, where IDUs report being denied or overcharged for syringes at pharmacies.

**Methods:**

Trained "mystery shoppers" attempted to buy a 1 cc insulin syringe according to a predetermined script at all retail pharmacies in three Tijuana neighborhoods. The same pharmacies were surveyed by telephone regarding their syringe sales policies. Data on purchase attempts were analyzed using basic statistics to obtain an objective measure of syringe access and compared with data on stated sales policies to ascertain consistency.

**Results:**

Only 46 (28.4%) of 162 syringe purchase attempts were successful. Leading reasons for unsuccessful attempts were being told that the pharmacy didn't sell syringes (35.3%), there were no syringes in stock (31.0%), or a prescription was required (20.7%). Of 136 pharmacies also surveyed by telephone, a majority (88.2%) reported selling syringes but only one-third (32.5%) had a successful mystery shopper purchase; the majority of unsuccessful purchases were attributed to being told the pharmacy didn't sell syringes. There was similar discordance regarding prescription policies: 74 pharmacies said in the telephone survey that they did not require a prescription for syringes, yet 10 of these pharmacies asked the mystery shopper for a prescription.

**Conclusions:**

IDUs in Tijuana have limited access to syringes through retail pharmacies and policies and practices regarding syringe sales are inconsistent. Reasons for these restrictive and inconsistent practices must be identified and addressed to expand syringe access, reduce syringe sharing and prevent HIV transmission.

## Background

Injection drug users (IDUs) are at high risk of infection with HIV, hepatitis C (HCV) and other blood-borne pathogens transmitted by sharing syringes and other injection equipment. Globally, almost 20% of the world's 15.9 million IDUs are infected with HIV [1] and in some studies HCV prevalence among IDUs is >90% [2].

Transmission of these pathogens can be prevented by eliminating syringe sharing among IDUs. This requires that sterile syringes be available at appropriate times and in sufficient quantities to supply a sterile syringe for each injection. In most settings, IDUs' avenues for obtaining sterile syringes are limited to syringe exchange programs (SEPs) and pharmacies. SEPs have proven effective in reducing syringe sharing [3], but the number of these programs - and the overall number of syringes they distribute - is not sufficient to provide IDUs with a sterile syringe for each injection. In Latin America, for example, only 5 of 20 countries are known to have implemented SEPs, which serve only approximately 2% of the region's IDUs [4].

Pharmacies can provide a more comprehensive and convenient source of syringes for IDUs, as they generally exceed SEPs in number of locations and hours of operation. In some areas, however, pharmacy-based syringe access is hampered by laws requiring a prescription for purchase. Allowing purchase without a prescription has been shown to increase the number of syringes sold and reduce sharing among IDUs. In the United States, for example, where syringe access laws vary by state, Connecticut and Minnesota saw substantial increases in the number of IDUs who reported pharmacy purchase of syringes and decreases in syringe sharing following repeal of syringe prescription laws [5,6]. Similarly, in New York City, an Expanded Syringe Access Demonstration Program (ESAP) was associated with a significant increase in the proportion of IDUs who obtained syringes from pharmacies, and these IDUs were less likely than others to report syringe sharing [7].

Unfortunately, even where the "laws on the books" allow syringe purchase without a prescription, IDUs still encounter barriers to purchase. U.S.-based studies have obtained objective measures of IDUs' ability to purchase syringes using the "mystery shopper" method, in which study personnel enter pharmacies and attempt to purchase a sterile syringe according to a predetermined script. These studies have documented refusal rates of 31-59% in areas where syringe purchase without a prescription is legal [8-12].

In Mexico, where syringe purchase without a prescription is also legal, IDUs report being refused or overcharged for syringes at retail pharmacies and link these refusals directly with syringe sharing [13,14]. In Tijuana, a northwestern Mexico border city adjacent to San Diego, California, 59% of IDUs report receptive syringe sharing in the past 6 months and HCV prevalence is 96% [15,16]. HIV prevalence among Tijuana's male IDUs, female IDUs and female IDUs who engage in sex work is 4%, 10%, and 12%, respectively [17,18] and as many as one in 125 persons aged 15-49 in the city are estimated to be HIV-positive [19]. We undertook this study to obtain an objective measure of barriers to pharmacy-based syringe purchase among IDUs in Tijuana and assess the need for pharmacy-based HIV prevention interventions.

## Methods

### Study setting

Tijuana, Mexico has a population of 1.6 million [20] and is situated on a major illicit drug trafficking route that brings heroin, methamphetamine and other illicit drugs northward into the United States [21]. Drugs that do not make it over the U.S. border are sold plentifully and cheaply in Tijuana [22,23] which is the site of a growing drug using population; lifetime illicit drug use prevalence in Baja California, the state where Tijuana is located, is 9.3% compared to a national prevalence of 5.2% [24], and there are an estimated 10,000 IDUs in the city [25]. Tijuana is also home to a thriving cross-border market for legal pharmaceuticals, which are sought by U.S. consumers for their relatively cheap prices. As a result, pharmacies are ubiquitous in Tijuana and are particularly concentrated near the U.S. border, where multiple pharmacies commonly exist on the same city block.

### Human subjects

The study protocol was reviewed and approved by the Ethics Board of the Tijuana General Hospital and the Human Research Protections Program of the University of California, San Diego. A waiver of consent for pharmacy personnel was granted on the grounds that the protocol met the requirements of 45 CFR 46.116(d); the research was determined to be of minimal risk to participants (e.g., involvement was limited to normal sales activities, no personally identifiable information was collected), the waiver would not adversely affect the subjects' rights or welfare, and the research could not practicably be carried out without the waiver. Further, our primary Mexican collaborator (R.L.) assured that study findings would be presented to state and local health departments and the local pharmacy association for dissemination to Tijuana pharmacies after completion of the study.

### Data collection

#### Mystery shopper syringe purchases

Between April 2006 and April 2007, data on locations of injection drug use were collected at baseline from participants in Proyecto El Cuete, a longitudinal study of 1,056 IDUs in Tijuana. Based on these data, we identified three *colonias *(neighborhoods) as the most common areas of injection drug use in Tijuana: Zona Norte, Zona Centro and Zona Rio, all of which are located near the U.S. border and cover an area of approximately 2.6 square miles. Using a list of registered pharmacies provided by the health department of Baja California and street maps from Proyecto El Cuete as a guide, our study staff went street-by-street to create a validated list of all retail pharmacies in these three *colonias*.

During August and September, 2009, each pharmacy was randomly assigned to one of four "mystery shoppers," i.e., two male and two female study staff wearing casual dress common to IDUs. Mystery shoppers were trained to enter their assigned pharmacies and attempt to purchase a single 1 cc insulin syringe according to a predetermined script, which insured uniformity across purchases. Shoppers were instructed to pay ≤ 10 pesos per syringe, which was the median price of syringes purchased at retail pharmacies based on self-reported baseline data from Proyecto El Cuete, and not to negotiate with pharmacy staff during the purchase attempt. They were also instructed not to disclose their identity or the purpose of their visit to pharmacy staff at the time of the purchase attempt. Syringe purchases were attempted between the hours of 8:00 am and 6:00 pm and were conducted both on weekdays and weekends.

In all cases, the mystery shopper was driven to the target pharmacy in a car with a driver and second study staffer who waited nearby during the purchase attempt. After leaving the pharmacy the mystery shopper immediately returned to the car and was debriefed there by the second study staffer, who recorded information from the purchase attempt on a data collection form. This allowed us to immediately record the details and outcome of the syringe purchase attempt without requiring that data be recorded inside the pharmacy, thus protecting the nature and purpose of the purchase attempt from immediate disclosure to pharmacy staff. Data collected included the date and time of the attempt; number of other shoppers in the store; characteristics of the staff person from whom the syringe was requested (e.g., sex, approximate age); syringe price; whether the purchase was successful; and any additional details the shopper could provide regarding their interaction with pharmacy staff. Information from the paper data collection form was subsequently entered into a database using Microsoft Excel.

#### Telephone survey

Between September 2009 and February 2010, the same pharmacies were contacted by telephone by a female project staffer. The person who answered the phone was asked whether they sold 1 cc insulin syringes and, if so, how much they cost and whether a prescription was required for purchase. These data were recorded on a standardized form and entered into the same Excel database for analysis. Although the telephone survey was conducted after the mystery shopper visits were completed, the mystery shopper study and its results had not yet been shared with health departments, pharmacy associations or pharmacy staff; therefore, the risk of the telephone survey responses being influenced by knowledge of the mystery shopper data collection was minimal.

### Data analysis

Data from mystery shopper purchase attempts were tabulated to determine the percentage of successful syringe purchases and reasons for failed purchases. We also identified factors associated with purchase outcome by comparing the characteristics of successful versus unsuccessful purchase attempts using Wilcoxon rank-sum tests for continuous variables and the Pearson's chi-square test for categorical variables. Data from the telephone survey were similarly tabulated to determine the percentage of pharmacies that reported selling syringes and the percentage that required a prescription for purchase. These results were then compared with data from the mystery shopper purchase attempts to identify discrepant findings.

## Results

Overall we identified 189 retail pharmacies in the three targeted *colonias *and completed purchase attempts at 164 of them. The other 25 pharmacies were excluded because either the pharmacy had closed since the validated list was completed (n = 17) or it was determined not to be an eligible retail pharmacy at the time of the purchase attempt (n = 8; e.g., sold botanicals only, pediatric medications only). Of the 164 purchase attempts, one was excluded because the mystery shopper varied from the predetermined script and one was excluded due to missing data, leaving a total of 162 eligible purchase attempts included in our analysis.

### Mystery shopper syringe purchases

Only 46 (28.4%) of the 162 eligible mystery shopper purchase attempts were successful. The median price per syringe purchased was 7 pesos (IQR: 5-10). Table 1 compares the characteristics of successful and unsuccessful purchase attempts. There were no statistically significant differences between the pharmacies where syringes were successfully purchased and those where the purchase attempt was unsuccessful; however, female mystery shoppers were more likely to have a successful purchase outcome than male shoppers, with marginal significance (p = 0.058).

**Table 1:** Characteristics of successful and unsuccessful retail pharmacy syringe purchase attempts (N = 162)

	Successful (%) N = 46	Unsuccessful (%) N = 116	P-value
Mystery shopper sex			
Male	19 (41.3)	67 (58.7)	.058
Female	27 (58.7)	49 (42.2)	
Median number of pharmacy staff who waited on mystery shopper (IQR)	1 (1-2)	1 (1-2)	.406
Sex of staff person 1			
Male	23 (50.0)	54 (47.0)	.727
Female	23 (50.0)	61 (53.0)	
Approximate age of staff person 1	31 (25-45)	35 (27-44)	.260
Sex of staff person 2			
Male	7 (46.7)	11 (40.7)	.710
Female	8 (53.3)	16 (59.3)	
Approximate age of staff person 2	40 (30-55)	31 (25-44)	.109
Median number of customers within 10 feet of shopper	1(0-2)	0 (0-1)	.154
Number of customers in store			
< 5	43 (97.7)	113 (98.3)	.825
5-10	1 (2.3)	2 (1.7)	
> 10	0	0	

**Figure 1:**
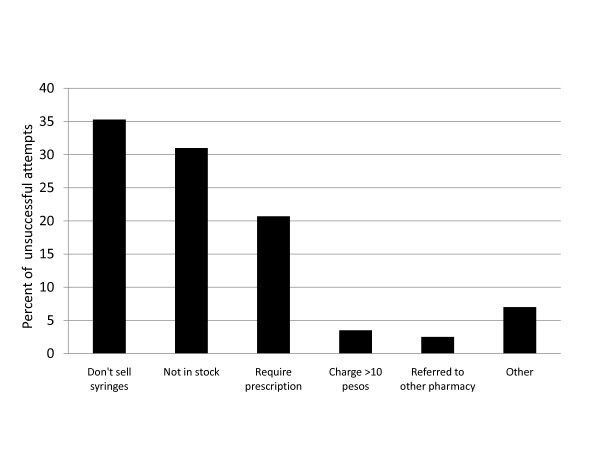
**Reasons for unsuccessful syringe purchase attempts (N = 116)**.

Figure 1 presents the reasons for unsuccessful purchase attempts. One-third (35.3%) of the pharmacies told the mystery shopper they did not sell syringes and another one-third (31.0%) said they had no syringes in stock. The remaining purchase attempts failed either because the pharmacy requested a prescription (20.7%), charged more than the maximum established price of 10 pesos (3.5%; price range 12-15 pesos), referred the mystery shopper to another pharmacy (2.5%), or for some other reason (7.0%; e.g., told that syringes were only sold in packages of 10, only sold 10 cc syringes).

In some cases, the mystery shoppers provided additional comments regarding their experiences during the syringe purchase attempt. In five cases, the shopper noted that although their purchase attempt was unsuccessful the person who waited on them treated them kindly, as if this experience were out of the ordinary. In two cases in which the purchase attempt was successful, the shopper noted that the seller told them to "take care" or "be careful." There were two cases in which the shopper specifically reported that the person who waited on them was angry or upset, and two others where they reported being actively observed (e.g., "[They] looked at me from head to toe, studied me [before they said] 'I can't sell it to you without a prescription.'"). In three other failed attempts the shopper reported being completely ignored by the pharmacy staff (e.g., "[They] continued watching television, didn't even look at me.").

In six cases, the mystery shopper reported lack of knowledge about the pharmacy policy regarding syringe sales or that pharmacy staff disagreed about whether to sell them the syringe. These interactions were described as follows:

• "He/she was going to sell it to me but a voice from behind the wall told him/her that I needed a prescription."

• "The guy was going to help me but they girl said they had run out...she was shaking her head at the guy as if to say 'No, don't sell.'"

• "One (the one that was selling) said yes, but then asked the boss who said they didn't have any."

• "The lady was going to sell to me, the man wasn't. 'We don't have syringes because we don't have a fridge for the insulin.'"

• "He/she had to call a supervisor by radio to ask if he/she could sell it to me or not. [The supervisor said]...not without a prescription."

• "One said that with a prescription and the other one said that there was no problem. In the end it was sold."

### Telephone survey

Overall, 136 (84.0%) of the 162 pharmacies also completed the telephone survey; of the 26 pharmacies who did not complete the survey, a majority (73.1%) could not be contacted because they did not have a listed phone number or the phone number was incorrect or out of service. Only one pharmacy refused to answer questions. More pharmacies that completed the telephone survey had a successful mystery shopper purchase attempt than those that did not complete the survey (30.9% vs. 15.4%) but the difference was not statistically significant (p = 0.11). The median reported price per syringe was 6 pesos (IQR: 5-9 pesos), slightly lower than the median price of 7 pesos charged to the mystery shoppers.

A comparison of the telephone survey and mystery shopper outcomes is provided in Table 2. Overall, 120 (88.2%) of the 136 pharmacies surveyed reported selling syringes. Of these 120 pharmacies, 32.5% had a successful mystery shopper outcome and 67.5% an unsuccessful outcome. In the latter cases where pharmacies reported selling syringes but did not sell one to the mystery shopper, the most common reason for the failed purchase attempt was being told that the pharmacy did not sell syringes (38.3%). Conversely, there were three pharmacies that reported not selling syringes in the telephone survey but sold a syringe to the mystery shopper.

**Table 2:** Comparison of telephone survey and mystery shopper outcomes

	**Phone survey outcomes**			
		**Sells syringes**	**Doesn't sell syringes**	**Total**
	
	**Successful**	39 (32.5)	3 (18.8)	42 (30.9)
	**Unsuccessful**	81 (67.5)	13 (81.3)	94 (69.1)
	**Total**	120 (88.2)	16 (11.8)	136 (100.0)
**Mystery shopper outcomes**				
		**No prescription**	**Requires prescription**	**Total**
	
	**No prescription**	64 (86.5)	13 (59.1)	77 (70.6)
	**Requires prescription**	10 (13.5)	22 (62.9)	32 (29.4)
	**Total**	74 (67.9)	35 (32.1)	109* (100.0)

*11 of the 120 pharmacies that reported selling syringes in the telephone survey did not give a definitive answer regarding their prescription policies

There was also a high level of discordance regarding prescription requirements, as shown in Table 2. Overall, 35 pharmacies (32.1%) said in the telephone survey that they require a prescription for syringe purchase even though Mexican law does not require it; however, 13 of these pharmacies sold a syringe to the mystery shopper without a prescription. Seventy-four pharmacies (61.7%) said they did not require a prescription for syringe purchase but 10 of these pharmacies refused the mystery shopper purchase due to lack of a prescription. The remaining 11 pharmacies surveyed did not give a definitive answer regarding whether they required a prescription for syringe purchases, with seven stating (without being prompted by the caller) that their decision to ask a customer for a prescription depended on what the customer looked like. None of these 11 pharmacies asked the mystery shopper for a prescription and 5 had successful mystery shopper purchases.

## Discussion

To our knowledge, this is the first published mystery shopper study of syringe access conducted in a developing country and the first anywhere to compare mystery shopper outcomes with a concurrent telephone survey. We documented a very low level of success in purchasing sterile syringes at retail pharmacies in Tijuana, Mexico, and a high level of discordance between stated pharmacy syringe sales policies and those experienced by the mystery shoppers.

The low mystery shopper success rate in this study corroborates IDU reports of substantial barriers to pharmacy-based syringe purchase in Tijuana. In qualitative studies, IDUs have linked these barriers directly to risky injection practices, including syringe sharing and scavenging through medical and household waste for used syringes [13,14]. A quantitative study of IDUs in Proyecto El Cuete similarly demonstrated an independent association between experiencing barriers to pharmacy-based syringe purchase and receptive syringe sharing, syringe reuse, and a higher number of lifetime abscesses [14]. In light of the mounting evidence regarding restrictive syringe sales practices in Tijuana and their direct contribution to risky injection behaviors, structural interventions are needed to modify these sales practices.

This study provides preliminary insights into the reasons for restrictive syringe sales practices in Tijuana. First, we found high levels of discordance between stated pharmacy syringe sales practices and mystery shopper outcomes. The fact that practices experienced by the mystery shoppers were more restrictive than those stated in the telephone survey suggests pharmacies are less likely to sell syringes to suspected IDUs. Our mystery shoppers were study staffers who, although not current drug users, had a history of injection drug use and for the purposes of the study dressed in a manner consistent with IDUs in the area; it is thus reasonable to believe that they were suspected of injection drug use. Further, seven pharmacies in our telephone survey willingly stated that their decision to request a prescription for syringe purchase hinged on the appearance of the customer. IDUs in Tijuana have spoken at length with us about the perceived role of appearance in their attempts to purchase syringes, and we have demonstrated an independent association between homelessness - which influences the ability of IDUs to maintain the cleanliness of their person and clothing - and encountering barriers to syringe purchase [14]. These findings indicate that suspecting a person of injection drug use is a motivating factor for pharmacy staff in denying syringe purchase in Tijuana.

U.S. studies have identified a number of reasons why pharmacies deny syringes to suspected IDUs. These include business considerations including worries regarding store theft, the security of pharmacy staff and customers, and increased drug use and discarding of used syringes near the pharmacy [26-34]. Individual attitudes of pharmacy staff also play a role; these include negative attitudes toward drug use and drug users, concerns that distributing syringes increases drug use and the belief that selling syringes is not appropriate for pharmacists in their role as health care professionals [26,28,30,31,34]. Studies that incorporate interviews with pharmacy owners, pharmacists and clerks in Tijuana are needed to determine whether these factors influence syringe sales decisions and identify other factors amenable to intervention. These studies constitute the next phase of our research activities in Tijuana.

One of these other factors may be misunderstanding of the laws regarding pharmacy syringe sales in Mexico. Almost one-third of the pharmacies interviewed by telephone said they require a prescription for syringe purchase. It is possible that pharmacy management and staff do not have an accurate understanding of the laws governing syringe sales in Mexico. Alternatively, it is possible that, for reasons that remain unclear, they feel the need to obtain a prescription despite what the law allows. In qualitative interviews with IDUs, they posited that pharmacies fear retribution from police if they are caught selling syringes to IDUs [14]. None of the pharmacies in this study cited fear of police to the mystery shopper or telephone interviewer, but prior studies in Tijuana by our research team have found high rates of arrest for possession of sterile syringes among IDUs, even though possession of these syringes is allowed under Mexican law [35]. If fear of police is indeed a factor in pharmacy syringe sales practices then police behavior would need to be targeted as part of any pharmacy-based intervention.

Regardless of their reasons for requesting a prescription, it is clear that pharmacies' prescription policies are not applied consistently. Our study found discordance between pharmacy prescription policies reported in the telephone survey and policies encountered by the mystery shoppers at the same pharmacies. As expected, most of these discrepancies went in one direction, with pharmacies reporting not requiring a prescription but asking the mystery shopper for one anyway. However, we also identified pharmacies that sold syringes to the mystery shopper despite a stated policy of requiring a prescription. Further, our mystery shoppers reported encountering disagreements among pharmacy staff regarding whether or not to sell the shopper a syringe; these disagreements may explain some of the discrepancies we encountered in comparing our mystery shopper and telephone survey findings, as the outcome may have depended greatly upon which pharmacy staffer waited on the mystery shopper or answered the survey call. Interventions that seek to bring pharmacies into compliance with Mexican laws allowing over-the-counter syringe sales will need to ensure that these policies are understood and implemented consistently across the staff within each pharmacy.

Finally, the overall rate of successful purchases achieved in this study was lower than the success rates achieved by U.S.-based studies employing a similar methodology [8-12]. Although our findings cannot be extrapolated to other developing countries - or even to other regions in Mexico - they raise the possibility that access to sterile syringes through retail pharmacies in developing countries where syringes are legally available without a prescription may be more limited than in developed countries with the same policies. This possibility should be investigated by researchers in developing countries where IDUs constitute a substantial proportion of new HIV cases.

U.S.-based mystery shopper studies have documented significant differences in outcomes depending on whether a retail pharmacy is a chain or independent establishment. Because we were not able to determine the chain/independent status of several pharmacies while developing our validated pharmacy list, we did not include this variable in our study. We also identified only a very small number of pharmacies that attempted to overcharge the mystery shoppers for syringes, which was inconsistent with our prior qualitative findings that overcharging is common [13,14]. This may be attributed to the fact that, for reasons of staffing and safety, we did not conduct any purchase attempts after 6:00 pm. Given that IDUs have reported higher likelihood of overcharging in the late evenings and early mornings, our findings may underestimate the frequency of overcharging. IDUs have also reported a higher likelihood of overcharging when they appear to be in opiate withdrawal, which our mystery shoppers clearly were not.

## Conclusions

We documented a substantial difference between the "laws on the books" that govern syringe sales in Mexico and the actual sales practices of retail pharmacies in Tijuana. A clearer understanding of what motivates these sales practices is needed. Structural interventions that build upon this knowledge should be prioritized among public health efforts to expand syringe access and reduce transmission of HIV and other blood-borne pathogens among IDUs.

## Competing interests

The authors declare that they have no competing interests.

## Authors' contributions

RP, RL and MG conceived of and designed the study. GM and KB contributed to the development of the data collection instruments, sampling strategy and study protocol. PR and BR carried out the data collection. RP analyzed the data and drafted the manuscript. All authors read and approved the final manuscript.

## References

[B1] Mathers BM Degenhardt L Phillips B Wiessing L Hickman M Strathdee SA Wodak A Panda S Tyndall Touflik A Mattick RP 2007 Reference Group to the UN on HIV and Injecting Drug Use Global epidemiology of injecting drug use and HIV among people who inject drugs: a systematic review Lancet 2008 372 1733 1745 10.1016/S0140-6736(08)61311-2 18817968

[B2] Aceijas C Rhodes T Global estimates of prevalence of HCV infection among injecting drug users Int J Drug Policy 2007 18 352 358 10.1016/j.drugpo.2007.04.004 17854722

[B3] Committee on the Prevention of HIV Infection among Injection Drug Users in High Risk Countries, Board on Global Health, Institute of Medicine of the National Academies Preventing HIV Infection among Injecting Drug Users in High-Risk Countries: An Assessment of the Evidence 2007 Washington, DC: National Academies Press

[B4] Mathers BM Degenhardt L Ali H Wiessing L Hickman M Mattick RP Myers B Ambekar A Strathdee SA 2009 Reference Group to the UN on HIV and Injecting Drug Use HIV prevention, treatment, and care services for people who inject drugs: a systematic review of global, regional, and national coverage Lancet 2010 375 1014 1028 10.1016/S0140-6736(10)60232-2 20189638

[B5] Cotten-Oldenburg NU Carr P DeBoer JM Collison EK Novotny G Impact of pharmacy-based syringe access on injection practices among injection drug users in Minnesota, 1998 to 1999 J Acquir Immune Defic Syndr 2001 27 183 192 11404541 10.1097/00126334-200106010-00014

[B6] Groseclose SL Weinstein B Jones TS Valleroy LA Fehrs LJ Kassler W Impact of increased legal access to needles and syringes on practices of injecting-drug users and police officers - Connecticut, 1992-1993 J Acquir Immune Defic Syndr Hum Retrovirol 1995 10 82 89 7648290

[B7] Pouget ER Deren S Fuller CM Blaney S McMahon JM Kang SY Tortu S Andia JF Des Jarlais DC Vlahov D Receptive syringe sharing among injection drug users in Harlem and the Bronx during the New York State Expanded Syringe Access Demonstration Program J Acquir Immune Defic Syndr 2005 39 471 477 10.1097/01.qai.0000152395.82885.c0 16010172

[B8] Compton WM Horton JC Cottler LB Booth R Leukefeld CG Singer M Cunningham-Williams R Reich W Fortuin Corsi K Staton M Fink JL Stopka TJ Spitznagel EL A multistate trial of pharmacy syringe purchase J Urban Health 2004 81 661 670 15466847 10.1093/jurban/jth149 PMC3455919

[B9] Deibert RJ Goldbaum G Parker TR Hagan H Marks R Hanrahan M Thiede H Increased access to pharmacy sales of syringes in Seattle-King County, Washington: Structural and individual-level changes, 1996 versus 2003 Am J Public Health 2006 96 134 10.2105/AJPH.2003.032698 PMC1522120 16809607

[B10] Finkelstein R Tiger R Greenwald R Mukherjee R Pharmacy syringe sale practices during the first year of expanded syringe availability in New York City (2001-2002) J Am Pharm Assoc 2002 42 Suppl 2 S83 87 10.1331/1086-5802.42.0.s83.finkelstein 12489622

[B11] Koester SK Bush TW Lewis BA Limited access to syringes for injection drug users in pharmacies in Denver, Colorado J Am Pharm Assoc 2002 42 Suppl 2 S88 91 10.1331/1086-5802.42.0.s88.koester 12489623

[B12] Trubatch BN Fisher DG Cagle HH Fenaughty AM Johnson ME Nonprescription pharmacy sales of needles and syringes Am J Public Health 2000 90 1639 1640 10.2105/AJPH.90.10.1639 11030005 PMC1446366

[B13] Strathdee SA Fraga WD Case P Firestone M Brouwer KC Perez SG Magis C Fraga MA "Vivo para consumirla y la consume para vivir" ["I live to inject and inject to live"]: high risk injection behaviors in Tijuana, Mexico J Urban Health 2005 82 3 Suppl 4 iv58 73 16107441 10.1093/jurban/jti108 PMC2196210

[B14] Pollini RA Lozada R Gallardo M Rosen P Vera A Macias A Palinkas LA Strathdee SA Barriers to pharmacy-based siringe purchase among injection drug users in Tijuana, Mexico: a mixed methods study AIDS Behav 2010 14 679 687 10.1007/s10461-010-9674-3 20300820 PMC2865643

[B15] Strathdee SA Lozada R Pollini RA Brouwer KC Mantsios A Abramovitz DA Rhodes T Latkin CA Loza O Alvelais J Magis-Rodriguez C Patterson TL Individual, social, and environmental influences associated with HIV infection among injection drug users in Tijuana, Mexico J Acquir Immune Defic Syndr 2008 47 369 376 10.1097/QAI.0b013e318160d5ae 18176320 PMC2752692

[B16] White EF Garfein RS Brouwer KC Lozada R Ramos R Firestone-Cruz M Pérez SG Magis-Rodríguez C Conde-Glez CJ Strathdee SA Prevalence of hepatitis C virus and HIV infection among injection drug users in two Mexican cities bordering the US Salud Publica Mex 2007 49 165 172 17589770 10.1590/s0036-36342007000300001 PMC2743977

[B17] Strathdee SA Lozada R Ojeda VD Pollini RA Brouwer KC Vera A Cornelius W Nguyen L Magis-Rodriguez C Patterson TL Proyecto El Cuete Differential effects of migration and deportation on HIV infection among male and female injection drug users in Tijuana, Mexico PLoS One 2008 3 e2690 10.1371/journal.pone.0002690 18665250 PMC2467493

[B18] Strathdee SA Philbin MM Semple SJ Pu M Orozovich P Martinez G Lozada R Fraga M de la Torre A Staines H Magis-Rodríguez C Patterson TL Correlates of injection drug use among female sex workers in two Mexico-U.S. border cities Drug Alcohol Depend 2008 92 132 140 10.1016/j.drugalcdep.2007.07.001 17714888 PMC2213538

[B19] Brouwer KC Strathdee SA Magis-Rodríguez C Bravo-García E Gayet C Patterson TL Bertozzi SM Hogg RS Estimated numbers of men and women infected with HIV/AIDS in Tijuana, Mexico J Urban Health 2006 83 299 307 10.1007/s11524-005-9027-0 16736378 PMC2527171

[B20] Consejo Nacional de Población (CONAPO) De la población de México 2005-2050 http://www.conapo.gob.mx/index.php?option=com_content&view=article&id=36&Itemid=199 Accessed May 7, 2010.

[B21] National Drug Intelligence Center, U.S. Department of Justice National Drug Threat Assessment 2010 http://www.justice.gov/ndic/pubs38/38661/index.htm Accessed July 2, 2010.

[B22] Brouwer KC Case P Ramos R Magis-Rodríguez C Bucardo J Patterson TL Strathdee SA Trends in production, trafficking, and consumption of methamphetamine and cocaine in Mexico Subst Use Misuse 2006 41 707 727 (2006) 10.1080/10826080500411478 16603456 PMC2757051

[B23] Bucardo J Brouwer KC Magis-Rodriguez C Ramos R Fraga M Perez SG Patterson TL Strathdee SA Historical trends in the production and consumption of illicit drugs in Mexico: Implications for the prevention of blood borne infections Drug Alcohol Depend 2005 79 281 293 10.1016/j.drugalcdep.2005.02.003 16102372 PMC2196212

[B24] Instituto Nacional de Salud Pública Encuesta Nacional de Adicciones 2008 http://www.insp.mx/images/stories/INSP/EncNacAdi/Docs/ENA08_nacional.pdf Accessed May 18, 2011.

[B25] Magis-Rodríguez C Brouwer KC Morales S Hayet C Lozada R Ortiz-Mondragón Ricketts EP Strathdee SA HIV prevalence and correlates of receptive needle sharing among injection drug users in the Mexican-U.S. border city of Tijuana J Psychoactive Drugs 2005 37 333 339 16295018 10.1080/02791072.2005.10400528

[B26] Farley TA Niccolai LM Billeter M Kissinger PJ Grace M Attitudes and practices of pharmacy managers regarding needle sales to injection drug users J Am Pharm Assoc 1999 39 23 26 10.1016/s1086-5802(16)30411-9 9990183

[B27] Glanz A Byrne C Jackson P Role of community pharmacies in preventing AIDS among injecting drug misusers: findings from a survey in England and Wales BMJ 299 1076 1079 10.1136/bmj.299.6707.1076 PMC1837978 2511969

[B28] Lewis BA Koester SK Bush TW Pharmacists' attitudes and concerns regarding syringe sales to injection drug users in Denver, Colorado J Am Pharm Assoc 2002 42 Suppl 2 S46 51 10.1331/1086-5802.42.0.s46.lewis 12489615

[B29] Marks RW Hanrahan M Williams DH Goldbaum G Thiede H Wood RW Encouraging pharmacy sale and safe disposal of syringes in Seattle, Washington J Am Pharm Assoc 2002 42 Suppl 2 S26 27 10.1331/1086-5802.42.0.s26.marks 12489609

[B30] Singer M Baer HA Scott G Horowitz S Weinstein B Pharmacy access to syringes among injecting drug users: follow-up findings from Hartford, Connecticut Public Health Rep 1998 133 Suppl 1 81 89 (1998) PMC1307730 9722813

[B31] Taussig J Junge B Burris S Jones TS Sterk CE Individual and structural influences in shaping pharmacists' decisions to sell syringes to injection drug users in Atlanta, Georgia J Am Pharm Assoc 2002 42 Suppl 2 S40 45 10.1331/1086-5802.42.0.s40.taussig 12489614

[B32] Tsai R Goh EH Webeck P Mullins J Prevention of human immunodeficiency virus infection among intravenous drug users in New South Wales, Australia: the needles and syringes distribution programme through retail pharmacies Asia Pac J Public Health 1988 2 245 251 3179107 10.1177/101053958800200408

[B33] Wright-De Aguero L Weinstein B Jones TS Miles J Impact of the change in Connecticut syringe prescription laws on pharmacy sales and pharmacy managers' practices J Acquir Immune Defic Syndr Hum Retrovirol 1998 18 Suppl 1 S102 110 9663632 10.1097/00042560-199802001-00018

[B34] Blumenthal WJ Springer KW Jones TS Sterk CE Pharmacy student knowledge, attitudes, and beliefs about selling syringes to injection drug users J Am Pharm Assoc 2002 42 Suppl 2 SS34 39 10.1331/1086-5802.42.0.s34.blumenthal 12489613

[B35] Pollini RA Brouwer KC Lozada RM Syringe possession arrests are associated with receptive sharing in two Mexico-US border cities Addiction 2008 103 101 108 10.1111/j.1360-0443.2007.02051.x 18028520 PMC2214830

